# Three dimensional socket preservation: a technique for soft tissue augmentation along with socket grafting

**DOI:** 10.1186/1750-1164-6-3

**Published:** 2012-04-27

**Authors:** Gholam Ali Gholami, Maryam Aghaloo, Farzin Ghanavati, Reza Amid, Mahdi Kadkhodazadeh

**Affiliations:** 1 Dept. of Periodontics, Dental School, Shahid beheshti Medical University, Tehran, Iran; 2 Dept. of Periodontics, Dental School, Qazvin University of Medical Sciences, Qazvin, Iran; 3 Private Practice, Tehran, Iran

**Keywords:** Bone, Dental implant, Esthetic

## Abstract

**Background:**

A cursory review of the current socket preservation literatures well depicts the necessity of further esthetic considerations through the corrective procedures of the alveolar ridge upon and post extraction. A new technique has been described here is a rotational pedicle combined epithelialized and connective tissue graft (RPC graft) adjunct with immediate guided tissue regeneration (GBR) procedure.

**Results:**

We reviewed this technique through a case report and discuss it’s benefit in compare to other socket preservation procedures.

**Conclusion:**

The main advantages of RPC graft would be summarized as follows: stable primary closure during bone remodeling, saving or crating sufficient vestibular depth, making adequate keratinized gingiva on the buccal surface, and being esthetically pleasant.

## Introduction

Several studies have concerned the morphological alterations occurred in alveolar process as a consequence of tooth extraction, both vertically and in the width of the residual bone [1,2]. The resorption rate is a factor of the time since extraction [3]. The contour loss occurs at a more significant rate during the early post-extraction period, especially within the first six months. changes in the buccal alveolar bone plate result in a collapse of the alveolar process, especially in the maxillary bone [4]. The subsequent ridge deformity poses a challenge to the rehabilitation process due to the significant functional and esthetic problems especially in the anterior maxillary region [5,6]. Conservative atraumatic extraction and socket preservation techniques with different materials have been evolved and clinically implemented [7,8]. Recent preservation approaches tend mostly towards the regenerated bone quality as a prerequisite for gaining a proper implant site and less towards the topographic status of the edentulous ridge [9].

On the other hand, various techniques have been discussed to achieve proper soft tissue closure at immediate implant sites. Pedicle flap technique and coronally displaced flap are two of the most important relevant corrective approaches [10]. Also, the use of autogenous soft tissue grafts to seal extraction sites before or at the time of implant placement has been described through case reports [11]. Using punch technique in addition to epithelialized connective tissue graft is another option for covering the extraction socket. The success of this soft tissue punch technique depends on the gingival graft receiving adequate vascular supply to remain viable [12]. The survival rate of the transplanted graft tissue depends on the nourishment from the organizing blood clot beneath the graft and from the marginal soft tissue in contact to it [13]. Esthetic results, restorative manipulation, soft tissue maturation, plaque control, protection from bacterial aggression and regular maintenance are enhanced when keratinized tissue surrounds the implant-supported prosthesis [14]. So, soft tissue rehabilitation should be considered as necessary as hard tissue reconstruction during or after tooth extraction.

It seems that if graft is pedicle and self-supplied with sufficient blood, a significantly higher survival rate would be expected. Then, it is necessary to seek a new soft tissue graft approach which will meet both keratinized tissue augmentation and blood supply demands. A review of the current socket preservation literatures depicts the necessity of further esthetic considerations through the corrective procedures of the alveolar ridge upon and post extraction.

## Methods

### Case description

#### Patient

**Figure 1:**
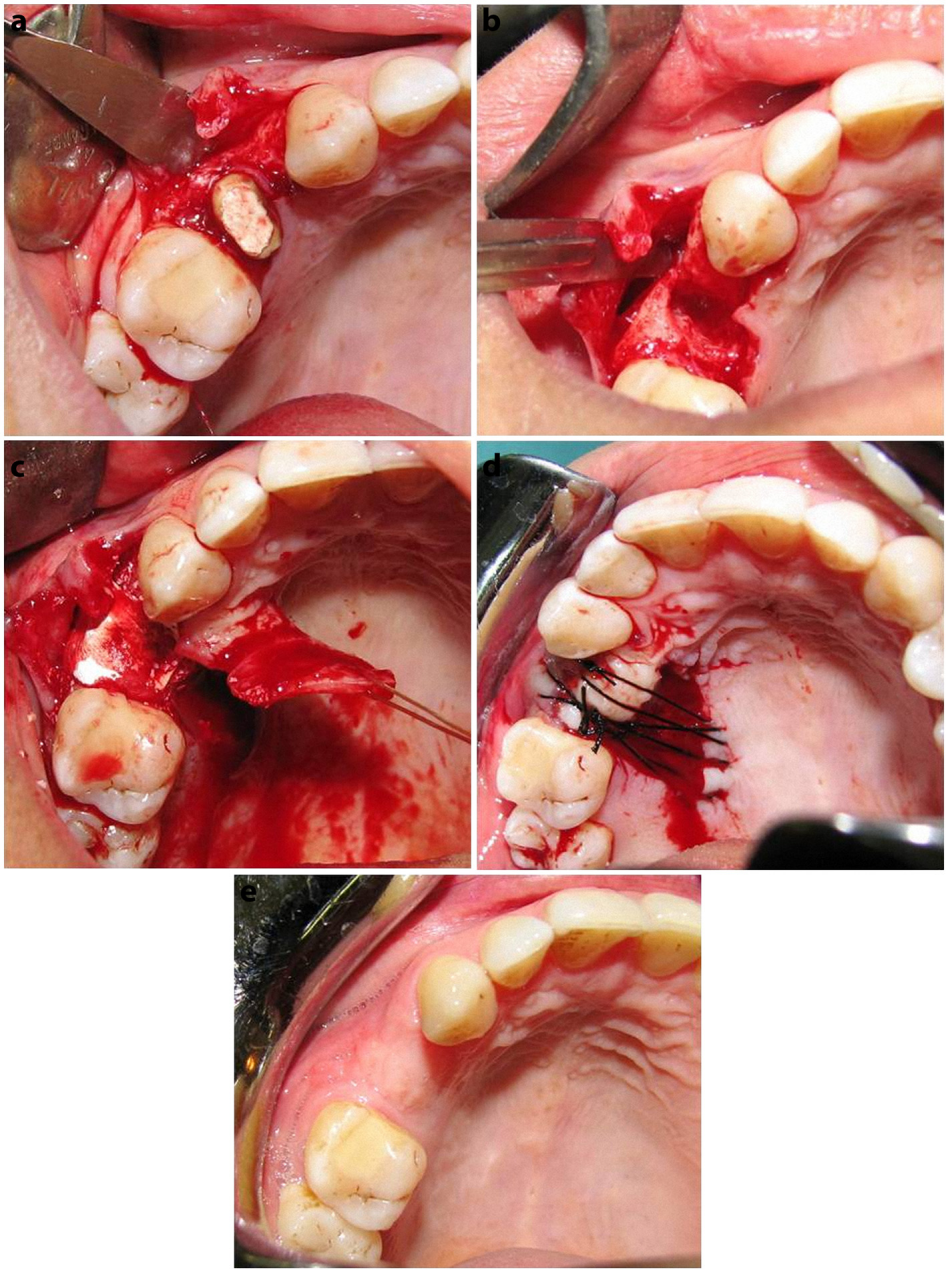
** a) Full thickness flap was raised 3 mm beyond the mucogingival junction without releasing incisions with adequate mesial and distal extension to form a pocket**. This pocket will later cover the graft ending. Buccal bone had adequate height and width hence no need for buccal overbuilding and membrane placement. b) clinical situation immediately after root extraction. c) The socket was filled with DFDBA. A partial thickness pedicle flap was raised. The flap was placed in position and the de-epithelialized ending was placed into the buccal pocket and- d) Sutured. Dressing was placed on the graft and the denuded donor site. e) Same patient three months post-operatively. Notice the vestibular height and the esthetics of the graft site

A 35 years old female with congenital missing right maxillary first premolar and tilting of the adjacent teeth towards the edentulous area was referred to our clinic. The right maxillary second premolar was crown-less and was endodontically hopeless. Based on the wax up analysis, the best potential implant site was determined to be mesial to the remained root (Figure 1-a). It was then decided to preserve the extraction socket for proper implant therapy. Written informed consent was obtained from the patient for publication of this report and any accompanying images.

#### Technique

**Figure 2:**
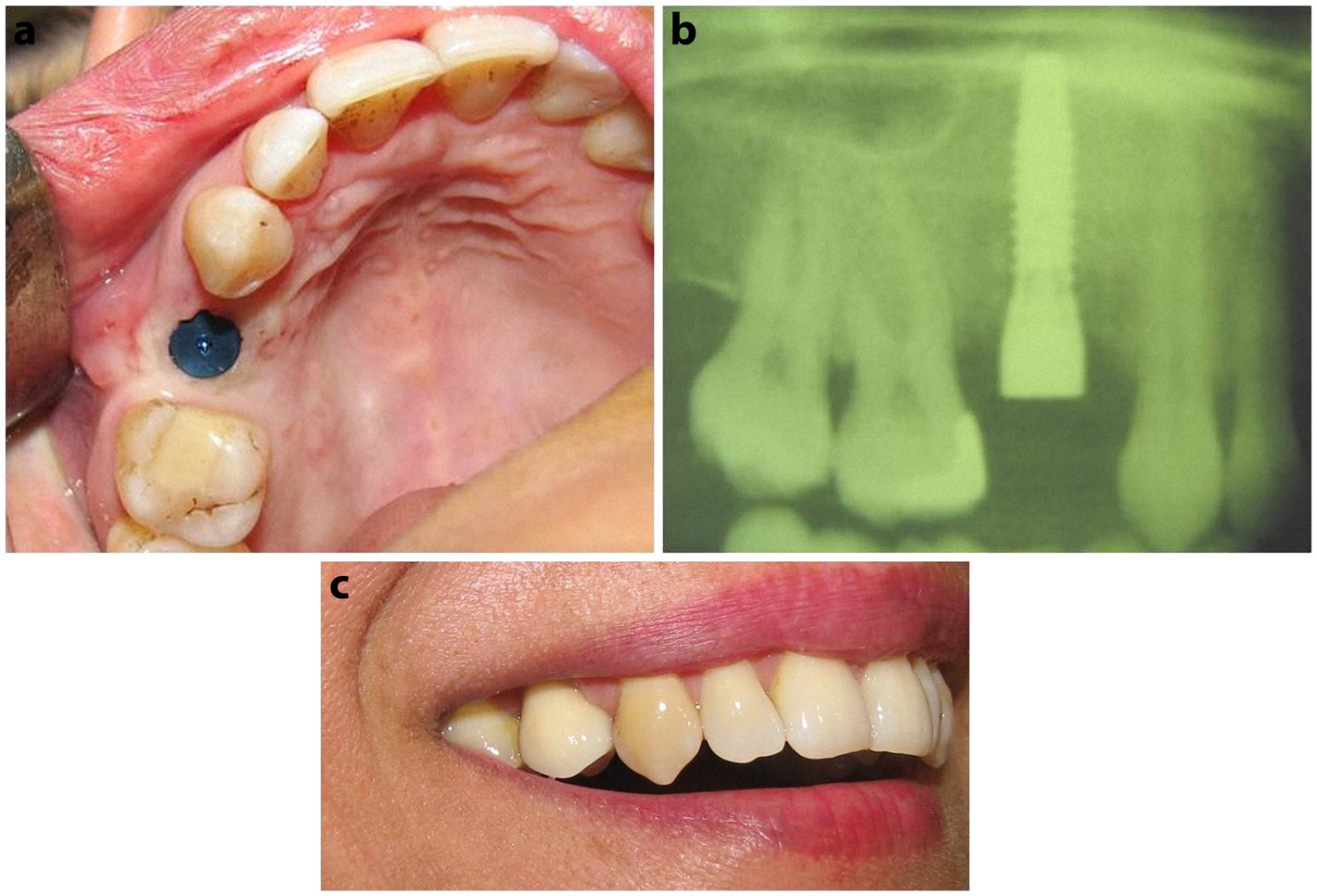
** a) One stage implant placement without flap elevation. b**) Conventional radiographic view. **c**) Patient appearance during smile.

The remained root was extracted with minor violation to the structures in vicinity (atraumatic conservative approach). A mucoperiosteal flap was raised on the buccal side of the edentulous site (Figure 1-b). No releasing incisions were made. So, the mesial, distal, and apical extension of the flap followed an envelope pattern. The flap was raised at least 3 mm beyond the mucogingival junction through the alveolar mucosa in order to form a pocket. This pocket would be used for saving the connective tissue segment of graft. Socket was thoroughly debrided and irrigated with normal saline and the socket bony walls were decorticated using a round bur. The socket was then filled with decalcified Freeze-Dried Bone Allograft (DFDBA, Iranian Tissue Bank, Iran) and covered by a collagen membrane (Bio-Gide, Geistlich , Switzerland). A rectangular pedicle flap was raised on the same palatal side (Figure 2-c). The pedicle placed mesially to the socket and in the anterior of the flap avoiding greater palatal foramen as much as possible. The graft width was equal to the mesiodistal width of the socket and its length was two times more than the buccolingual width of the socket. The free ending of the palatal flap was completely de-epithelialized so it can be easily placed into the buccal pocket. The rest of the flap, which was close to the base, was left epithelialized and full thickness. Hence, a Rotational Pedicle Combined (epithelialized and connective tissue) graft (RPC graft) established. Cut back incisions were made so the flap will be rotated freely. The flap was rotated and the de-epithelialized ending was placed into the buccal pocket without suturing. Continues cross suture placed over the graft to stabilize and make a scaffold for clot over palatal donor side (Figure 2-d).

## Results and discussion

### Definite treatment

The patient was an implant supported prosthesis candidate. Reevaluation six months later showed sufficient bone volume and soft tissue maturation ready for implant placement. Bone width in the implantation site was 6 mm and the height was 15 mm. A punch whole was made in the potential implant site and fixture was placed without flap reflection (Figure 2-a and b). Four months later, the implant was successfully loaded (Figure 2-c).

Socket preservation techniques have mostly concerned hard tissue augmentation and prevention of ridge collapse. Van der Weijden et al had a review of data about alveolar bone dimensional changes of extraction sockets in human. They calculated the data from 12 publications and reported an average 3.87 mm reduction in width and 1.67 mm bone loss in height after normal remodeling [15]. Soft tissue dimensional changes, however, has less been paid attention to through the literatures. The presence of adequate keratinized gingiva is of paramount importance in the long term survival of an implant, whereas a noticeable amount of buccal keratinized attached gingiva is coronally positioned to gain enough wound closure (GBR prerequisite) in conventional socket preservation techniques [16]. This is usually associated with complications like decreased vestibular depth, lack of adequate keratinized gingiva on the buccal side of the implant, and the coronal displacement of the mucogingival line which places a red alveolar mucosa tissue instead of a pink attached gingival [17]. This color mismatch poses an esthetic challenge and necessitates a second corrective graft surgery for buccal soft tissue augmentation. This also will be associated with a couple of post-operative problems e.g. scar tissue formation, compromised blood supply to the area, and excess costs [18]. The RPC graft technique presented in this paper will not only satisfy our need to prevent bone resorption but will also meet the soft tissue augmentation and preservation demands.

The main **advantages** of above mentioned technique could be summarized as:

· The epithelialized part of the pedicle graft covers the socket orifice and the de-epithelialized part is placed under the buccal flap. This not only ensures the proper closure of the socket, but also enhances the contour on the buccal side and contributes to the blood supply of the graft site. Underlying palatal graft will enhance the quality of the covering attached gingiva.

· The healing process then occurs through first intention on the socket orifice. It must be mentioned that one early clinical concern in all kinds of socket preservation procedures was wound premature opening [19].

· There is no need for releasing incisions since buccal flap is aimed to form a pocket.

· There is no need to coronally position the buccal flap. The mucogingival line level is then preserved as normal and buccal flap may even be positioned apically in an attempt to correct inadequate vestibular depth.

· Placing the non-epithelialized part of the pedicle graft under the buccal pocket beyond the mucogingival junction will make amends for the probable future buccal collapse.

· Due to adequate blood supply within the pedicle graft, socket inclusions will be nourished not only through socket walls but also from the flap. This will increase the chance of graft survival and enhance the future osseointegration in the potential implant site.

**Figure 3:**
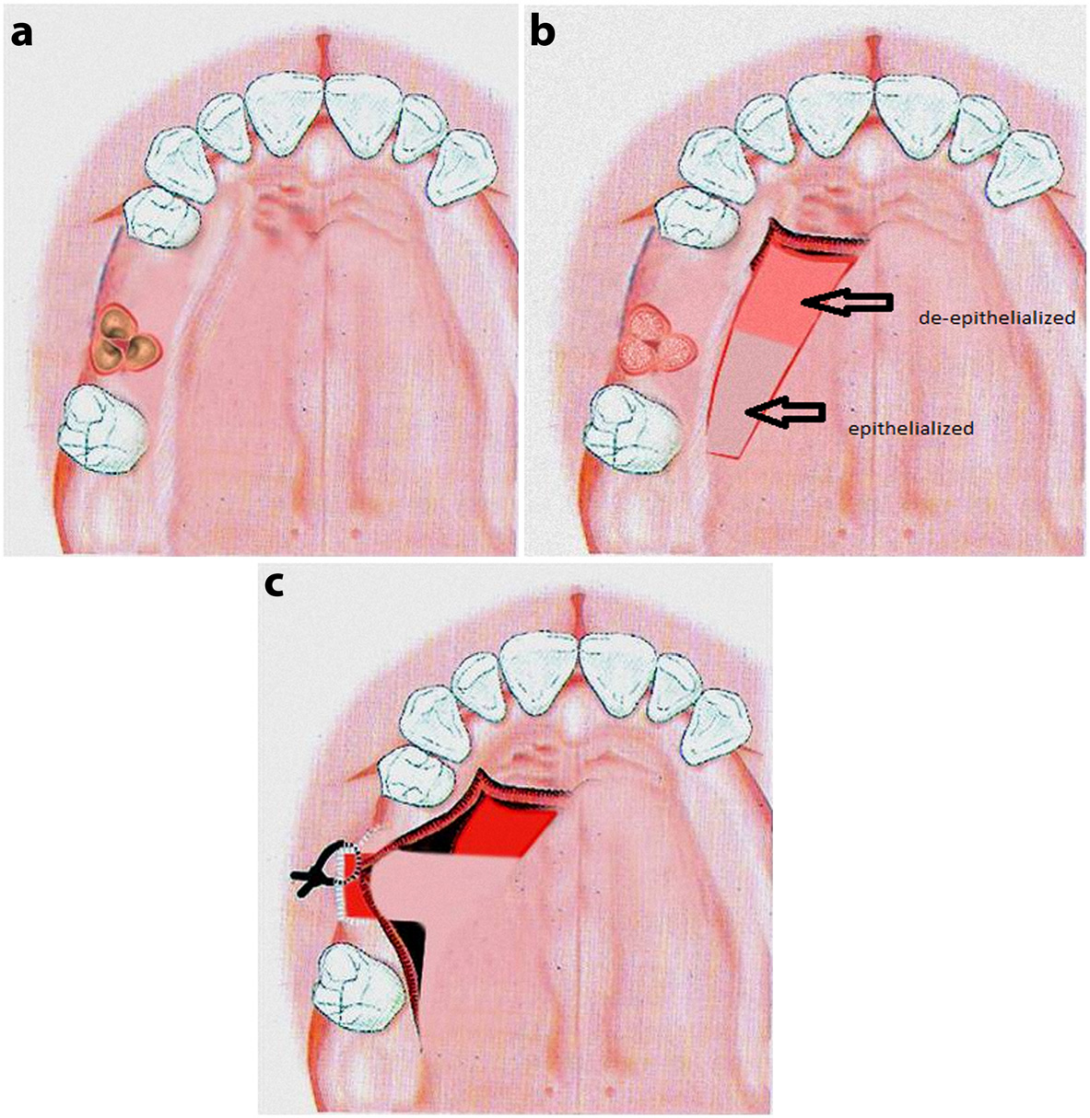
** a) Schematic view of RPC graft. b**) Flap design. **c**) Suturing.

· This technique will not only provide sufficient functional masticatory mucosa but also will provide maximum buccal soft tissue augmentation (Figure 3).

· Future buccal depression results from the tissue collapse (which is an inevitable consequence of remodeling) will be prevented due to the overbuilding of soft tissue in the area.

· This technique along with the harvest of a thick graft in the mesial and distal donor pedicle, papillae generation could be achieved to some extent in patients where gingival papillae have become flattened.

**Figure 4:**
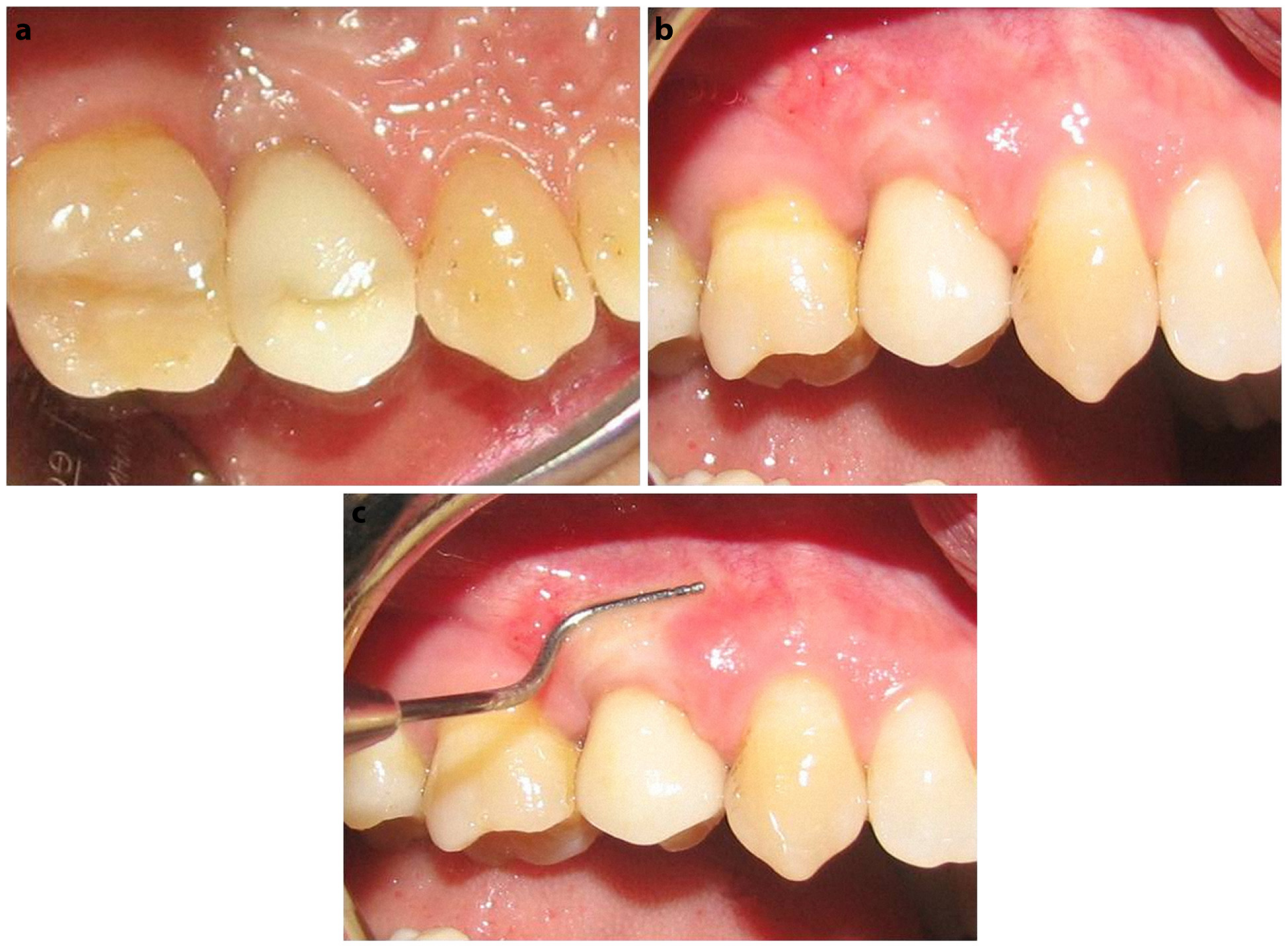
** a) Palatal view of implant-supported prosthesis after 6 month loading. b**) Buccal view. **c**) Excellent soft tissue with a sufficient band of attached/ keratinized tissue achieved by RPC graft in one session.

· This technique is associated with esthetically pleasant outcomes since the connective tissue graft have the advantage of color matching to the overlying tissue (Figure 4).

· Adequately extended incisions along with the application of cut-back incisions will allow free rotation of the flap and its passive placement on the expected area. There is then no need to suture the graft in place. When needed, the flap end may be sutured using resorbable material to the underlying connective tissue.

· This technique usually does not need a coronal repositioning of the buccal flap and thus no mucogingival junction displacements would be expected [20]. The present (prior to extraction) attached gingiva will then be preserved and the papillae around the expected implant will be of sufficient height.

The possible **modifications** of this technique are as follows:

· Full thickness flap is suggested in cases where buccal marginal bone needs overbuilding and partial thickness flap is recommended where adequate intact (minimum of 2 mm) buccal table is present post-extraction [21].

· In the cases where the buccal table needs overbuilding, the socket width will be covered with a resorbable collagen membrane after bone graft is placed. However, if the buccal plate is intact, there is no need for buccal table overbuilding or membrane application.

· The socket may be filled with resorbable bone substitutes like DFDBA or semi-resorbable bone substitutes like nano-bone.

· There is then no need to suture the graft in place. When needed, the flap end may be sutured using resorbable material to the underlying connective tissue.

Based on the type of bone substitute used, a 3 to 6 month bone healing period should be considered prior to implantation [22]. Also, there is no need for another flap during implant placement and punch technique (as for the present case) will be sufficient. All techniques with high predictability and proper esthetic outcome would be selected in socket management procedures [23,24].

## Conclusion

Socket preservation procedures used widely to manage the tissue dimensional alterations after tooth removal. These techniques considered as predictable procedures to reduce the need for extensive bone augmentation operations in implant dentistry. There are different socket/ridge preservation techniques with different outcomes. Also, there is no evidence to support the superiority of one specific technique over another. Our recommended technique named RPC graft would be useful in high esthetic demand cases due to its ability to reconstruct hard and soft tissue, simultaneously.

## Competing interests

The authors declare that they have no competing interests.
